# A noticeable difference? Productivity costs related to paid and unpaid work in economic evaluations on expensive drugs

**DOI:** 10.1007/s10198-015-0685-x

**Published:** 2015-04-16

**Authors:** Marieke Krol, Jocé Papenburg, Siok Swan Tan, Werner Brouwer, Leona Hakkaart

**Affiliations:** 1 Department of Health Policy and Management, Erasmus University, P.O. Box 1738, 3000 Rotterdam, The Netherlands; 2 Institute for Medical Technology Assessment, Erasmus University, Rotterdam, The Netherlands; 3 St. Willibrordus, Curacao

**Keywords:** Productivity costs, Indirect costs, Economic evaluation, Systematic review

## Abstract

Productivity costs can strongly impact cost-effectiveness outcomes. This study investigated the impact in the context of expensive hospital drugs. This study aimed to: (1) investigate the effect of productivity costs on cost-effectiveness outcomes, (2) determine whether economic evaluations of expensive drugs commonly include productivity costs related to paid and unpaid work, and (3) explore potential reasons for excluding productivity costs from the economic evaluation. We conducted a systematic literature review to identify economic evaluations of 33 expensive drugs. We analysed whether evaluations included productivity costs and whether inclusion or exclusion was related to the study population’s age, health and national health economic guidelines. The impact on cost-effectiveness outcomes was assessed in studies that included productivity costs. Of 249 identified economic evaluations of expensive drugs, 22 (9 %) included productivity costs related to paid work. One study included unpaid productivity. Mostly, productivity cost exclusion could not be explained by the study population’s age and health status, but national guidelines appeared influential. Productivity costs proved often highly influential. This study indicates that productivity costs in economic evaluations of expensive hospital drugs are commonly and inconsistently ignored in economic evaluations. This warrants caution in interpreting and comparing the results of these evaluations.

**Electronic supplementary material:**

The online version of this article (doi:10.1007/s10198-015-0685-x) contains supplementary material, which is available to authorized users.

## Introduction

The development of new and expensive health care technologies has increased the pressure on national health care budgets. Hence, there is a growing focus on whether new interventions offer value for money in terms of cost-effectiveness. This may especially be the case for very expensive interventions with an expected high budget impact, such as newly developed drugs administered in a hospital setting (e.g. new chemotherapies for cancer patients).

In order to determine the relative cost-effectiveness of new expensive interventions, many countries prescribe conducting a health economic evaluation. In an economic evaluation the health effects of two or more treatments are compared to their respective costs. Most countries that prescribe economic evaluations have formulated national health economic guidelines stipulating how these evaluations should be conducted. National guidelines are likely (and intended) to influence how economic evaluations are conducted in practice. One important aspect in such guidelines is the perspective the evaluation should take. Economic evaluations adopting a health care perspective include costs falling on the health care budget only, whereas economic evaluations adopting a societal perspective aim to include all relevant effects and costs, regardless of who bears the costs and who receives the benefits. Approximately half of the national guidelines on the ISPOR ‘pharmacoeconomic guidelines around the world’ website [1] prescribe taking a health care perspective (at least for the base case scenario) and the other half a societal perspective or a health care *and* societal perspective. However, most countries prescribing a health care perspective allow presenting additional cost-effectiveness scenarios that include broader societal costs, such as productivity costs. England and Wales, where the National Institute for Health and Care Excellence (NICE) operates [2] and New Zealand, were noticeable exceptions: those explicitly forbid including productivity costs in any of the presented analyses.

In the health economic literature, adopting a societal perspective is often advocated [3–7]. However, it is certainly not an undisputed choice [8].^1^ This lack of consensus regarding the appropriate perspective has likely contributed to the differences between national health economic guidelines. Interesting developments in this context are the likely changes in the United Kingdom (UK) where a shift to a value based pricing system is foreseen. Within the new system, economic evaluations should be conducted from the societal perspective instead of the currently applied health care perspective [9], which implies a major change in standpoint. Given the fact that the UK is one of the leading countries in performing and using economic evaluations in health care decision-making, this change may lead to more economic evaluations taking a societal perspective.

An important question is how such a difference in perspective could potentially affect decision-making. This obviously depends on the additional cost categories included in the analysis when performed from a societal perspective and their relative magnitude. The most influential cost category in that context may be productivity costs. Productivity costs can be defined as “costs associated with production loss and replacement costs due to illness, disability and death of productive persons, both paid and unpaid” [10]. Productivity costs can be quite influential on final outcomes of economic evaluations. For instance, in economic evaluations of treatments for depression, such costs, on average, reflect more than half of total costs, often strongly influencing incremental costs and, in turn, cost-effectiveness [11]. The inclusion of productivity costs (and the choice of perspective) thus can influence the allocation of scarce health care resources across diseases and patients if the latter is—at least to some extent—determined by incremental cost-effectiveness.

Even though half of the national health economic guidelines prescribe a societal perspective and productivity costs and savings can be substantial, previous studies suggest that, depending on the types of interventions and economic evaluations studied, not more than 8–31 % of economic evaluations actually include productivity costs related to paid work [11–13]. The inclusion of production loss related to unpaid labour seems even less common, although this has rarely been studied [13]. If productivity costs (related to both paid and unpaid labour) are indeed often ignored in economic evaluations, it is important to understand why this is the case and how final outcomes are influenced by ignoring these costs. It has been suggested that the choice regarding inclusion of productivity costs may sometimes be strategically driven by their expected impact on cost-effectiveness outcomes [11]. However, the exclusion of productivity costs may also be related to more pragmatic issues. In the case of expensive hospital drugs, for instance, it may be that productivity costs have a relative small impact on outcomes, for instance due to very high medical costs and (regarding productivity costs related to paid work) the relatively high age of patients. Under such circumstances, omitting productivity costs (or applying a health care perspective) potentially would not affect final cost-effectiveness ratios substantially. This, however, has never been investigated to our knowledge.

Therefore, this study sought to answer the question whether inclusion of these costs in expensive drugs studies is influential, in other words, whether they have a substantial impact on cost-effectiveness outcomes. Moreover, it aimed to determine whether cost-effectiveness studies of expensive drugs administered in a hospital setting normally include productivity costs related to paid and unpaid work. In addition, we aimed to explore how productivity costs were calculated in economic evaluations, i.e. which methodology was used to estimate productivity costs. Finally, we explored potential reasons for excluding productivity costs from the economic evaluation, such as countries’ health economic reimbursement submission guidelines as well as age and health status of the studies’ patient populations.

To meet the study objectives, we conducted an extensive systematic review of economic evaluations of 33 distinct expensive drugs administered in a hospital setting.^2^ The effect of including productivity costs on the cost-effectiveness outcomes was assessed by investigating those studies that included productivity costs and evaluating the impact of these productivity costs on final results.

Before describing and discussing the methods and results of our systematic review, in the Background Section we first discuss productivity costs and the potential explanations for the poor inclusion of productivity costs in economic evaluations of health care interventions.

## Background

Commonly, four categories of costs are distinguished in health economic evaluations: direct costs within health care, direct costs outside health care, indirect costs within health care and indirect costs outside health care [6, 7]. This latter category includes productivity costs which are an important cost-category and are widely recognised as real societal costs and potentially influential [13–15]. Nevertheless, the scarce available evidence suggests that these costs remain excluded from the majority of actual economic evaluations [11–13, 16]. Factors contributing to the neglect of productivity costs in economic evaluations can relate to the principles on which the economic evaluation is based, or pragmatic considerations throughout the execution of the economic evaluation. Pragmatic factors that contribute to ignoring productivity costs in economic evaluations could be a lack of time, data, research experience [17], or a (perceived) lack of relevance. Moreover, the lack of standardization of productivity cost methodology is likely to be of influence. Recently, some papers were published aimed at increasing standardization in this area [18–21].

### Relevance of including productivity costs in economic evaluations

Following ‘the rule of reason’, which states that if costs “…are trivially small or do not differ across regimens, their inclusion will have little effect on the final results of an analysis, and they may therefore be omitted at the analyst’s discretion” [6], productivity costs may be seen as irrelevant in several circumstances.

First, if productivity is not affected by some treatment, including productivity related costs would be superfluous, such as with some treatments aimed at very mild conditions or treatments aimed at very severe conditions in which patients are fully impaired without expectation of returning to paid or unpaid work (which may be the case for some patients receiving very expensive drug treatments in the hospital). In most cases, however, whether health interventions will affect patients’ productivity is difficult to predict.

Second, following to the rule of reason, productivity cost inclusion would be irrelevant if productivity is unchanged relative to the comparator (i.e. if productivity changes are similar in both the intervention and comparator groups), since productivity costs will then not change incremental cost-effectiveness ratios. For most interventions, determining up front whether productivity costs in both study arms will be equal is difficult, and excluding productivity costs on this ground is thus debatable.

Third, and also related to the rule of reason, productivity costs related to paid work may also be seen as less relevant for economic evaluations of interventions targeted at people not of working age. For instance, if most of the patients receiving an intervention are above retirement age, productivity losses related to paid work may be negligible. Note, however, that this is not the case for losses related to unpaid work. Treatments targeted at very young patients could affect future productivity, rendering productivity costs an important factor. Whether such effects are considered important to economic evaluations also depends on the valuation method chosen: related costs or savings should be included if using the human capital approach [22] but not if using the friction cost approach [23].

A final rationale for ignoring productivity costs in line with the rule of reason might be the expectation that these costs are not very influential if direct costs are relatively high. This might be the case in economic evaluations of (very) expensive drugs, especially when these drugs are administered in a (costly) inpatient setting. When, as a rule, productivity costs have little effect on cost-effectiveness outcomes in economic evaluations of expensive hospital drugs, they might be excluded a priori, limiting the burden to patients, and saving time and other resources. If the costs are influential (as they are in some areas [11, 24, 25]), ignoring productivity costs up front could lead to sub-optimal decision-making.

### Ethical concerns regarding the inclusion of productivity costs in economic evaluations

Next to questions on the relevance of productivity costs in some situations, the inclusion of productivity costs may lead to equity concerns, because their inclusion may favour reimbursement of health interventions targeted at the working population [26, 27]. If such interventions produce substantial societal savings by improving productivity levels, including productivity costs may result in more favourable cost-effectiveness outcomes than when similar interventions are used in less productive populations (e.g. very young or elderly). However, it is questionable whether ignoring the existence of costs and savings outside the health care sector is the proper solution to such ethical concerns, since it denies decision makers the opportunity to make well-informed decisions and balance potential savings with the equity implications of their decisions. Nevertheless, equity concerns are explicitly mentioned in some national health economic guidelines stipulating how to conduct economic evaluations for reimbursement of health interventions. For instance, in Australia [28] and New Zealand [29] the health economic guidelines prescribe a health care perspective rather than a societal perspective, based on equity arguments.

A number of reasons highlighted above may affect the inclusion of productivity costs in economic evaluations of expensive hospital drugs. We therefore set out to review the literature in order to investigate this further, as highlighted next.

## Methods

### Review of economic evaluations of expensive hospital drugs

We performed an extensive systematic review of all 33 drugs on the Dutch ‘expensive drug list’ in June 2009 to identify any economic evaluation. Table 1 presents the drugs and some examples of which diseases the drugs on the list are prescribed for. This list was chosen as the basis for the review, since drugs on this list pose a considerable burden on the health care drug budget. Although the Dutch list was used as a basis, drugs on this list pose a substantial burden on the health care budget in other Western countries as well. See for instance the paper of Hofman et al. [30] about future drug expenditure in hospitals in the United States. A drug is only eligible for placement on this list if the drug costs are very high (total drug expenses exceed a certain threshold) and the drug is administered within a hospital setting. Until recently, for drugs on the list, hospitals (normally subject to a yearly fixed budget) received additional financing in order to remove financial barriers which might stop patients from receiving these drugs [31].

**Table 1:** Pharmaceuticals included in the review (Dutch expensive hospital drug list June 2009)

Drug name	Example of prescription area
Docetaxel	Breast cancer, lung cancer, prostate cancer
Irinotecan	Colon cancer
Gemcitabine	Bladder cancer, breast cancer, lung cancer
Oxaliplatin	Colorectal cancer
Paclitaxel	Bladder cancer, ovarian cancer, melanoma
Rituximab	Leukemia, lymphomas
Infliximab	Ankylosing spondylitis, Crohn’s disease, rheumatoid arthritis
Intravenous immunoglobulin	Autoimmune diseases
Trastuzumab	Breast cancer
Botulin toxin	Several types of spasm
Verteporfin	Macular degeneration
Doxorubicin liposomal	Leukemia, several types of cancer
Vinorelbine	Lung cancer, breast cancer
Bevacizumab	Breast cancer, colorectal cancer, lung cancer
Pemetrexed	Pleural mesothelioma
Bortezomib	Multiple myeloma
Omalizumab	Asthma
Ibritumomab	Non-Hodgkin’s lymphoma
Pegaptanib	Macular degeneration
Alemtuzumab	Chronic lymphocytic leukemia, multiple sclerosis
Palifermin	Leukemia, lymphomas
Drotrecogin-alfa	Severe sepsis
Natalizumab	Crohn’s disease, multiple sclerosis
Cetuximab	Colon cancer
Ranibizumab	Macular degeneration
Abatacept	Rheumatoid arthritis
Voriconazole	Invasive aspergillosis, invasive candidiasis
Methyl aminolevulinate	Skin cancer
Panitumumab	Colorectal cancer
Anidulafungin	Invasive aspergillosis, invasive candidiasis
Caspofungin	Invasive aspergillosis, invasive candidiasis
Temsirolimus	Renal cancer
Temoporfin	Head and neck cancer

Note that this is not a complete list of diseases for which these drugs are prescribed

We used the Cochrane Library and PubMed databases with a publication date limit of January 1998 to June 2009.

Queries used for the database search were the drugs’ names and “cost” or “costs”. Inclusion criteria were (1) unique scientific articles in peer-reviewed journals in English, and (2) titles with terms or phrases such as cost(s), budget, economic, financial, price, money, dollar, economy, expenditure, pay, expense, fund, resource, reimbursement, consumption, or expensive. After excluding any reviews, we read abstracts of the remaining articles to determine if they were indeed economic evaluations. Finally, the full texts of the remaining articles were examined (with the exception of those unavailable in the Netherlands or British Library). Title and abstract searches were independently undertaken by two researchers. Full text examinations were carried out by two people in close collaboration.

### Inclusion of productivity costs

After identifying economic evaluations of expensive drugs, we investigated whether they included productivity costs related to paid or unpaid work and, if they did, how productivity losses were measured and valued. In order to do so we read the method sections of the papers and we examined the tables presenting the costs included in the individual studies.

Next, we explored whether the choice of including or excluding productivity costs is related to (1) age—working age of the patient population, (2) the patient population’s ability to work based on disease severity and (3) national health economic guidelines. Where possible we extracted the reported average or median age and the illness of the patients in the study populations. If the age of the patients was not reported in the paper, such as in several health economic modelling papers, we examined the paper reporting on the original clinical trial on which the modelling study was based. To explore whether age may explain the inclusion of productivity costs related to paid work, we assumed that at least a considerable part of a study population would be of working age if the average age of the study population was between 18 and 70. Subsequently, it was investigated whether inclusion or exclusion of productivity costs was related to the (estimated) health related ability to work, or the likelihood that at least a part of the patient population in individual studies would lose or regain the ability to work (in comparison to the control group). These estimations were based on a medical doctor’s expert opinion regarding the severity of disease of the patient populations as extracted from the papers (e.g., in the case of metastatic cancer it was assumed that (most) patients would not be able to perform paid work regardless of treatment). We also examined whether studies aligned with their national health economic guidelines regarding productivity costs. National and regional health economic guidelines were retrieved through the ISPOR ‘pharmacoeconomic guidelines around the world’ website [1]. Where possible, the original guideline documents were studied. If the guidelines were not available in English we followed the ISPOR “Key Features” pages. If guidelines were non-existent or not listed, they were labelled “unknown” and productivity cost exclusion could not be related to a recommended perspective.

### The impact of productivity costs on costs and cost-effectiveness

For the studies that included productivity costs, the percentage of total cost accounted for by productivity costs was calculated and reported. We then excluded productivity costs from the study’s incremental cost-effectiveness ratio(s) to analyse the impact of productivity costs on cost-effectiveness outcomes. We described whether inclusion of productivity costs led to change in incremental cost-effectiveness and the magnitude of the change. For cost-minimization studies (where incremental effects are insignificant) we examined only changes in incremental costs. All prices were adjusted to 2009 euros using the European Union Harmonized Indices of Consumer Prices published by Eurostat [32].

## Results

### Review

**Fig. 1:**
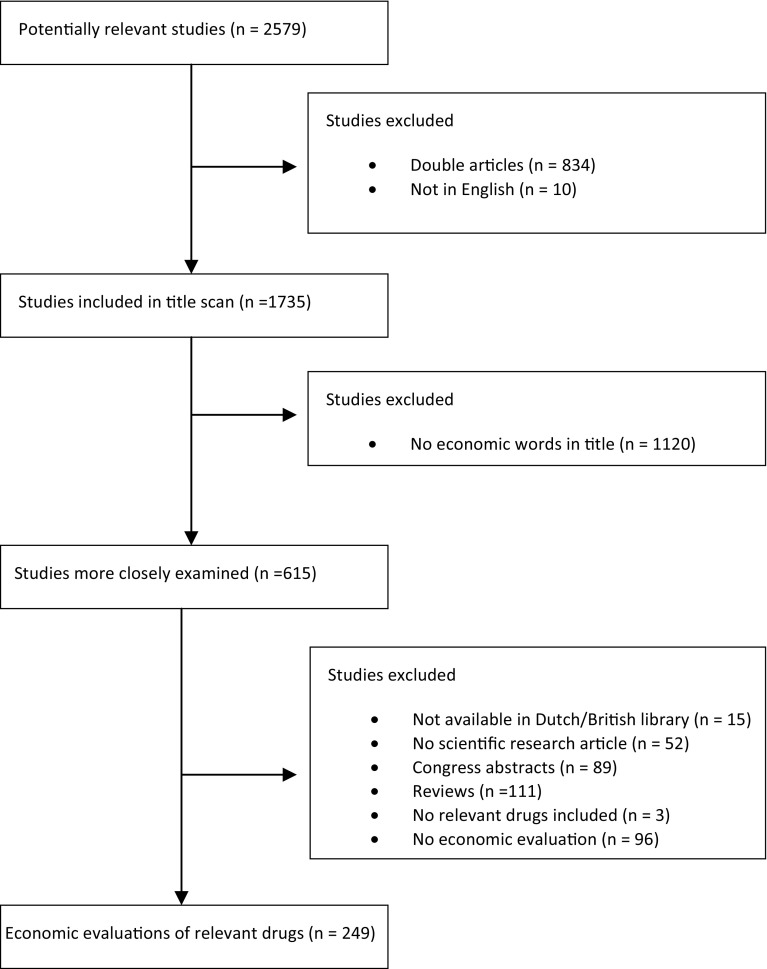
Flow diagram of the systematic literature review

The database search resulted in 2,157 articles in Pubmed and 422 in the Cochrane Library, 834 of which were doubles (Fig. 1). The number of doubles was quite high because many economic evaluations included more than one expensive drug and, as a consequence, appeared in several of the database searches of the individual drugs. Ten articles were not in English; 1,120 did not include an economic term or phrase in the title; 15 were not available in the Netherlands or British Library; 52 were not scientific research articles; 89 were congress abstracts; 111 were reviews; three did not evaluate one of the 33 drugs; and 96 could not be qualified as economic evaluations. This resulted in 249 economic evaluations of drugs from the Dutch expensive hospital drug list [31]. Of these, 22 (about 9 %) included productivity costs related to paid work and only one of these [33] additionally included productivity costs related to unpaid work. (See Online Resource for details of the studies.) Of the 22 economic evaluations including productivity costs, three were identified as cost minimization analyses (CMA); [34–36] the remaining 19 were cost-utility analyses (CUA), where effects are expressed in quality adjusted life years (QALYs). Eight of the 22 economic evaluations including productivity costs investigated treatments for rheumatoid arthritis, 5 were about ankylosing spondylitis, 3 were about breast cancer, 2 evaluated multiple sclerosis treatments, 1 was about colorectal cancer treatment, 1 was about ovarian cancer, one was about asthma and one studied sepsis.

### Inclusion of productivity costs: age, work ability and health economic guidelines

Inclusion (or exclusion) of productivity costs of all 249 economic evaluations was compared with the study-populations’ median or average age, the estimated work ability and the health economic guidelines of the respective countries. As can be seen in Table 2, most study-populations had an average age below 70 in both the studies including and excluding productivity costs. Based on a doctor’s opinion, the study populations of approximately one-third of the studies excluding productivity costs were expected not to be able to perform paid work regardless of treatment.

**Table 2:** Patients’ ages and health-related work ability

	Studies including PC (*n* = 22)	Studies excluding PC (*n* = 227)
Productive age (18–65)		
Yes (mean age 18–70)	19 (86 %)	163 (72 %)
No (mean age >70)	−	21 (9 %)
Unknown	3 (14 %)	42 (19 %)
Work ability based on severity of illness		
Likely to be able to work	21 (95 %)	146 (64 %)
Doubtful	1 (5 %)	8 (4 %)
Unlikely to be able to work	−	73 (32 %)

These estimations were based on a medical doctor’s expert opinion regarding the severity of disease of the patient populations

*PC* productivity costs

Based on national health economic guidelines, for 56 of the 249 economic evaluations, productivity cost inclusion was not allowed (i.e. studies from England, Wales or New Zealand) but four of the 56 included productivity costs anyway [35, 37–39]. In all four studies the incremental cost-effectiveness ratios (ICERs) decreased due to including productivity costs. Information on the countries the studies originated from and the perspective prescribed in the health economic guidelines of these countries can be found in Online Resource 1. Fourteen economic evaluations originated from countries for which no guidelines were available, only one of which included productivity costs [36]. In 74 economic evaluations, inclusion of productivity costs was mandatory according to the relevant health economic guidelines, but only 11 studies followed the rule. Despite this low level of inclusion, Table 3 shows that productivity costs were (as expected) more often included in evaluations originating from countries with guidelines prescribing inclusion. Only six of the 106 studies from countries in which productivity cost inclusion is allowed but not required included the costs [40–45].

**Table 3:** Productivity cost inclusion and national health economic guidelines

Perspectives in HE guidelines	Economic evaluations	Studies including PC	% inclusion
Health care for base case	56	4	7
PC not allowed in any scenario			
Health care for BC	74	4	5
PC allowed in additional scenarios			
Societal	41	7	17
Societal and health care	33	4	12
Societal or health care	32	2	6
Unknown	13	1	8
Total	249	22	9

*HE* health economic, *PC* productivity costs, *BC* base case

### Productivity costs methodology

Three [39, 46, 47] of the 22 studies including productivity costs provided no details on either the measurement of productivity costs (e.g. using patient questionnaires or literature estimates), or the valuation (the valuation approach used and the values attached to lost productivity).

Regarding the measurement of productivity costs, only seven studies [36–38, 42, 48–51] described actual data collection on productivity among the patient population, but none of them specified the productivity costs measurement instrument used. Two economic evaluations [34, 51] explicitly assumed that productivity would be relatively unaffected in all study arms; i.e., productivity costs were assumed not to vary. Consequently, in these studies, productivity costs were not quantified.

With regard to the valuation of lost work, ten of the 22 studies [33–36, 38, 40–42, 45, 49] used average (age- and gender-dependent) wage and employment rates to value lost working time. In one study [44] it was assumed (based on outcomes of a previous study) that productivity costs would be either equal to the direct costs or three times the direct costs. Another study [52] used employers’ annual labour costs. The remaining ten studies [37, 39, 43, 46–48, 50, 51, 53, 54] did not specify the values used. Three studies applied the friction cost approach [33, 34, 53]. These studies also applied the human capital approach. Only four of the 17 remaining studies [38, 43, 49, 54] explicitly mentioned applying the human capital approach. We assumed that studies not clearly specifying their method applied the human capital approach, which was indeed in line with their descriptions of productivity cost calculations. The one study that additionally included productivity costs related to unpaid work based the cost estimates on changes in household work and volunteer work [33]. The time spent on unpaid work was valued at the same rate as informal care (correcting for the costs already included for household help and informal care).

### Productivity costs inclusion: the proportion of total costs

We could not determine the proportion of total costs accounted for by productivity costs for 11 of the 22 articles due to a limited level of detail in the presented cost items. Four of these 11 [39, 40, 42, 47] did not provide specifications regarding the amount of productivity costs and seven only provided information on incremental productivity costs [37, 38, 43, 48, 49, 51, 52].

The remaining 11 articles provided information on the absolute amount of productivity costs. In these 11 studies, 37 estimates were available of productivity costs related to an intervention or comparator. Twenty-four of these estimates valued productivity costs according to the human capital approach [35, 36, 41, 44–46, 50, 54]; 13 applied both the human capital and the friction costs approach [33, 34, 53]. As expected, studies applying the human capital approach generated higher productivity costs, since in the friction cost approach the duration of inclusion of productivity loss is shorter. For studies applying the human capital approach, productivity costs on average comprised 45 % (range −106 to 83 %) of total costs. Productivity costs for the 13 estimates for which both the methods were applied were on average 24 % (range 4–38 %) of the total with the friction cost approach, and 44 % (range −106 to 80 %) with the human capital approach. For the study including unpaid labour costs [33], these costs were on average 0.7 % of the total, ranging from −25 % (i.e., led to savings) to 15 %, depending on the study arm.

### The impact of productivity costs on cost-effectiveness outcomes

**Fig. 2:**
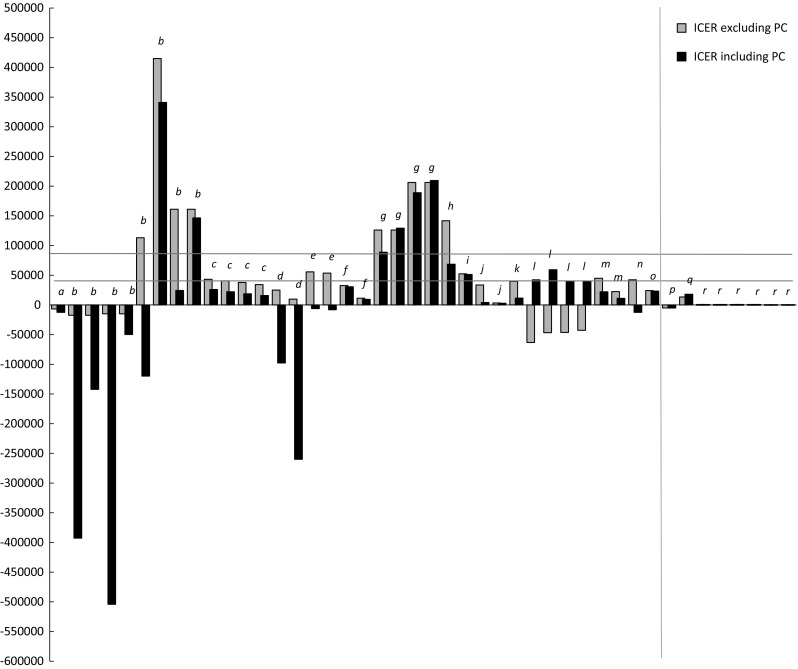
ICERS with and without productivity costs. ICERS from: (*a*) Lindgren et al. [46] (*b*) van den Hout et al. [33] (*c*) Davies et al. [41] (*d*) Kobelt, Sobocki et al. [48] (*e*) Kobelt et al. [37] (*f*) Kobelt et al. [43] (*g*) Boonen et al. [53] (*h*) Kobelt, Andlin-Sobocki et al. [43] (*i*) Kobelt, Eberhardt et al. [49] (*j*) Kobelt et al. [50] (*k*) Wong et al. [44] (*l*) Lidgren et al. [54] (*m*) Norum et al. [52] (*n*) Kobelt et al. [51] (*o*) Manns et al. [45] (*p*) Maniadakis et al. [36] (*q*) Walsh et al. [33] (*r*) Norum & Holtmon [34]

To study the impact of productivity costs on cost-effectiveness outcomes, the ICERs, including productivity cost, were recalculated after excluding these costs in the analyses. Since CMAs obviously do not present ICERs, given the assumption of equal effectiveness between comparators, we recalculated the cost differences after excluding productivity costs. Because only one study considered unpaid labour, we limited our calculations to paid labour only. Recalculation of cost-effectiveness outcomes was only possible for the 15 CUAs and three CMAs (representing a total of 36 ICERs and eight incremental cost calculations) specifying the amount of productivity costs, or the effect of productivity costs on the ICER. The change in incremental cost-effectiveness after excluding productivity costs is shown in Fig. 2. For all ICERs two bars are included. The first reflects the incremental cost-effectiveness in 2009 euros per QALY excluding productivity costs; the second reflects the incremental cost-effectiveness including productivity costs. The incremental costs of the three CMAs are presented on the right-hand side of the vertical line in Fig. 2. Productivity cost exclusion had little effect on the incremental costs in these studies. For six of the eight calculations, the incremental costs were close to zero with and without productivity costs. As a result, the bars representing the incremental costs in Fig. 2 are hardly visible.

We drew two fictive ICER thresholds in the figure for illustrative purposes: one at approximately €40,000 per incremental QALY in the region of the upper limit of the UK costs per QALY threshold [55] and one at €80,000, a suggested threshold for diseases with a very high burden of disease by the Dutch Council for Public Health and Health Care [56]. These threshold lines illustrate how productivity cost inclusion (exclusion) potentially affects decision-making for treatments where the ICER with productivity cost falls below (above) the threshold and the ICER without productivity costs above (below) the threshold. Note that we did not include uncertainty around the ICERS and the thresholds.

A comparison of ICERs with the inclusion and exclusion of productivity costs shows that ICERs increase due to inclusion of productivity costs in six out of 36 cases [53, 54]. In four of these cases the new treatment changed from cost-saving to cost-spending [54]. Including productivity costs led to a decrease of the IC(ER) in 30 cases [33, 37, 38, 41, 43–46, 48–53]. In six of these 30 cases the decrease caused the incremental costs to change from positive to negative, therefore turning the new treatment into a cost-saving intervention [33, 37, 48, 51].

Taking into account the fictive threshold of €40,000 per QALY, eight ICERs exceed the threshold when excluding productivity costs, while not exceeding the threshold when including productivity costs [33, 37, 41, 51, 52]. The other way around, three ICERs lie below the €40,000 threshold without productivity costs and exceed this threshold after including productivity costs [54]. In other words, in 11 of the 36 ICERs (31 %) including or excluding productivity costs would alter decision-making (if exclusively based on an ICER decision rule), based on a fixed €40,000 threshold. If we raise the threshold to €80,000, three ICERs exceed the threshold as a consequence of excluding productivity costs [33, 38]. With the €80,000 threshold, none of the ICERs is above the threshold including productivity costs and below the threshold after excluding these costs. With an €80,000 threshold, decision-making could alter in three of 36 cases (8 %).

## Discussion

This study investigated productivity cost inclusion and its impact on cost-effectiveness outcomes in economic evaluations of very expensive drugs. Moreover, the applied methodology regarding productivity cost measurement and valuation was assessed and possible explanatory factors for exclusion of these costs were explored. The results showed that productivity costs were excluded in over 90 % of the investigated economic evaluations. If productivity costs were included, the applied methods were mostly poorly reported.

Regarding the main objective of this study, determining whether productivity costs have a substantial impact on cost-effectiveness outcomes, when productivity costs were actually included this was clearly the case. Despite the high direct costs related to expensive drug treatment, productivity costs reflect a relatively high proportion of total costs and can strongly affect incremental cost-effectiveness ratios. With a fixed €40,000 threshold (somewhere in the region of the upper limit of the UK threshold [55]), decisions regarding reimbursement of expensive drugs could alter in almost one-third of the cases by including or excluding productivity costs in the cost-effectiveness analyses. In other words, the upcoming shift in perspective in the UK may have a strong impact on subsequent decision-making. The exact influence of productivity cost inclusion does not only depend on the methodology used to estimate productivity costs (and whether or not consumption is included [57]), it also depends on the decision framework. If the decision framework assumes a fixed budget and, hence, displacement of current health interventions, the productivity gains or losses related to these displaced activities could also be included in the evaluation [8].

Given the potential strong effects of productivity costs on final outcomes, productivity costs cannot be simply excluded in economic evaluations of expensive hospital drugs based on ‘the rule of reason’ introduced by Gold et al. [6]. Nevertheless, only one of the 249 identified economic evaluations included productivity costs related to both paid and unpaid work, and 21 (8 %) singly included productivity costs related to paid work. Such results indicate that productivity costs related to unpaid work rarely seem to play a role in cost-effectiveness calculations of expensive drugs administered in the hospital, and productivity costs related to paid work are ignored in the vast majority of studies. When we compare our findings to findings of previous studies [11–13], it seems that productivity cost inclusion (or rather exclusion) has not changed over recent decades.

A secondary objective of our study was to determine the extent to which productivity costs inclusion or exclusion is related to patients’ ages, severity of illness and countries’ health economic reimbursement submission guidelines. Assuming that an average study-population age below 70 implies that a considerable number of the patients are still of working age, age does not seem to explain the exclusion of productivity costs in economic evaluations of expensive hospital drugs. Health status may be of influence, however. In approximately one-third of the studies, the severity of illness of the patients could have been a reason for excluding productivity costs (related to paid work) up front. Moreover, our results indicate that health economic guidelines influence productivity cost inclusion or exclusion, but most guidelines leave room for judging *when* to include productivity costs and how to do so. Several studies did not seem to adhere to their national corresponding guidelines. Partly, this may be explained by some studies not being conducted for reimbursement submission purposes; however, such information was not presented in the papers. It is unclear what the consequences are of not adhering to national guidelines. Likely this differs between countries. Notably, in most of the studies we examined, clear information on how productivity costs were derived was lacking. It has been suggested that the decision to include productivity costs may be driven by strategic considerations regarding the expected influence on final outcomes, resulting in a selection-perspective bias [11]. Although too few studies in our review included productivity costs to be able to confirm this suggestion, the ICERs of every economic evaluation that included productivity costs against the relevant health economic guidelines decreased. The existence of a selection-perspective bias emphasizes the importance of standardizing economic evaluations. Given the large impact of productivity costs, transparency in measurement and valuation methods is paramount. Studies’ comparability, completeness and transferability would be served by consistent and preferably uniform inclusion of productivity costs, perhaps presented as a separate item. Inclusion also raises decision makers’ awareness of societal costs (or savings). If these costs are for any reason not included, it is important to justify their exclusion.

A limitation of our review is that by necessity we assessed the impact of productivity costs on cost-effectiveness outcomes based on the studies that actually included them. The amount of productivity costs in these studies may poorly reflect productivity costs in studies excluding these costs, especially if based on strategic considerations. Moreover, we were unable to determine how inclusion or exclusion of productivity costs related to unpaid work affects cost-effectiveness outcomes, since only one economic evaluation in our review considered unpaid work. Finally, we did not study or discuss the impact of the lack of scientific consensus regarding appropriate methods of measuring and valuing productivity costs on the exclusion of these costs. Numerous instruments, for example, can measure productivity costs (mainly related to paid work) but which instrument provides the most valid estimate is currently unknown. Estimates that vary substantially [58, 59] by using different instruments can result in a lack of confidence in the trustworthiness of productivity cost estimates. Given the fact that the studies in our review used a variety of measurement instruments, this is an important concern.

Next to measurement difficulties, the valuation of productivity costs (related to paid work) has been fiercely debated [23, 60–64]. The suitability of three valuation approaches has dominated the debates: the human capital approach, [22] the friction cost approach, [23] and the Washington panel approach [6]. For more information on the valuation approaches and the debates, see Tilling et al. [65] or Nyman [64]. The Washington panel approach received little theoretical and practical support, but lack of consensus on whether to apply the friction cost or the human capital approach translates to the use of both in practice [11, 13, 66].

## Conclusion

Productivity costs lead to noticeable differences in cost-effectiveness outcomes of economic evaluations of treatments with expensive hospital drugs. Despite the high direct costs related to the drugs, productivity costs reflect a non-negligible part of total costs when included and, therefore, a priori exclusion of productivity costs is not easy to defend when adopting a societal perspective. Ignoring productivity costs in economic evaluations of expensive hospital drugs without clear motive could imply ignoring important societal costs. That notwithstanding, productivity costs related to paid work are omitted in the majority of cases and productivity costs related to unpaid work are seldom included. The neglect of productivity costs is to some extent explained by the relevant national health economic guidelines prescribing a health care perspective, but the rationale for the majority of studies is unclear. We would argue that if productivity costs are ignored, motivation should be clear. Moreover, excluding productivity costs simply to comply with national guidelines does not render them less relevant to society or welfare improvement. Therefore, in countries prescribing a health care perspective a two-perspective approach, in which ICERs are presented from both a societal and health care perspective (as was done by some studies in this review), may be advisable [14].

## Electronic supplementary material

Below is the link to the electronic supplementary material.

Supplementary material 1 (DOC 164 kb)

## Footnotes

1 For further discussion on perspectives in economic evaluations see for instance Brouwer et al. [14], Claxton et al. [8], Johannesson et al. [3] and Jonssen [4].

2 These drugs were selected from the Dutch ‘expensive hospital drug list’ [31]. Until recently, hospitals in the Netherlands received additional financing for drugs placed on this list.
