# Supplementary material for: A noticeable difference? Productivity costs related to paid and unpaid work in economic evaluations on expensive drugs
Source: Eur J Health Econ. 2015 Apr 16;17(4):391–402. doi: 10.1007/s10198-015-0685-x (PMC4837201; doi:10.1007/s10198-015-0685-x)
Supplement: Supplementary file 1 — Supplementary material 1 (DOC 164 kb) [file 10198_2015_685_MOESM1_ESM.doc]

**A Noticeable Difference? Productivity costs related to paid and unpaid work in economic evaluations on expensive drugs**

European Journal of Health economics

**Authors and affiliation**

Marieke Krol ^a, b^, Jocé Papenburg ^a^, Siok Swan Tan ^a, b^, Werner Brouwer ^a, b^, and Leona Hakkaart ^a, b^

^a^ Department of Health Policy and Management, Erasmus University,

Rotterdam, The Netherlands

^b^ Institute for Medical Technology Assessment, Erasmus University, Rotterdam,

The Netherlands

**Correspondence**

*Marieke Krol*

*P.O. Box 1738*

*3000 DR Rotterdam*

*The Netherlands*

*T +31 10 408 8580*

*F +31 10 408 9081*

*krol@bmg.eur.nl*

**Online resource 1**

Studies including productivity costs (all costs in 2009 Euros)

| **Study** | **country** | **Type** | **PC inclusion** | **Perspective country** | **disease** | **age** | **Comparators** | **% PC/TC HCA** | **%PC/TC FCA** | **ICER** | **ICER-PC (or IC-PC)** | **ICER+PC (or IC+PC)** | **ICER change** |
| --- | --- | --- | --- | --- | --- | --- | --- | --- | --- | --- | --- | --- | --- |
| Maniadakis et al. 2009 | Greece | CMA | Days lost due treatment and AE, average daily earnings | Unknown | high risk colorectal cancer | 65.8 | XELOX | 0.25% |  | FOLFOX-XELOX | -5138 | -5210 | -70 |
|  |  |  |  |  |  |  | FOLFOX 6 | 0.57% |  |  |  |  |  |
| Bristow et al. 2007 | USA | CUA | Losses based on work-type adjusted hourly wages for full treatment period | Health care | stage III ovarian cancer | NS | IV/IV |  |  | IP/IV- IV/IV | NS | 30308 |  |
|  |  |  |  |  |  |  | IP/IV |  |  |  |  |  |  |
| Lindgren et al. 2009 | Sweden | CUA | NS | Societal | RA | 56.1 | RTX | 45.00% |  | RTX-TNF-inhibitor | -6875 | -12500 | -5625 |
|  |  |  |  |  |  |  | TNF-inhibitor | 45.00% |  |  |  |  |  |
| van den Hout et al. 2009 | NL | CUA | Average working hours minus worked hours, Age + sex-dependent hourly costs, FCA+HCA | Societal | recent-onset RA | 54.3 | Group 1 sequential monotherapy | 45.03% | 38.41% | 2-1 HCA | -17422 | -392772 | -375350 |
|  |  |  |  |  |  |  |  |  |  | 2-1 FCA | -17422 | -142225 | -124803 |
|  |  |  |  |  |  |  | Group 2 combination therapy | 22.20% | 31.57% | 3-1 HCA | -14967 | -504189 | -489222 |
|  |  |  |  |  |  |  |  |  |  | 3-1 FCA | -14967 | -49845 | -34878 |
|  |  |  |  |  |  |  | Group 3 combi+prednisone | -26.06% | 36.25% | 4-1 HCA | 113156 | -119830 | -232986 |
|  |  |  |  |  |  |  |  |  |  | 4-1 FCA | 414904 | 340931 | -73973 |
|  |  |  |  |  |  |  | Group 4 combi+IFX | -106.35% | 23.60% | 4-3 HCA | 161202 | 24305 | -136897 |
|  |  |  |  |  |  |  |  |  |  | 4-3 FCA | 161202 | 146541 | -14661 |
| Davies et al. 2009 | USA | CUA | PC based on proportion average earnings lost associated with lower HAQ score | Health care | early RA | NS | DMARD | 69.64% |  | IFX+MTX-DMARD | 43078 | 25971 | -17107 |
|  |  |  |  |  |  |  | IFX+MTX | 58.39% |  | ETN-DMARD | 40653 | 22201 | -18453 |
|  |  |  |  |  |  |  | ETN | 57.43% |  | ADA+MTX-DMARD | 37895 | 18785 | -19110 |
|  |  |  |  |  |  |  | ADA+MTX | 55.39% |  | ADA+MTX/ETN-DMARD | 34342 | 15804 | -18538 |
|  |  |  |  |  |  |  | ADA+MTX/ETN | 47.17% |  |  |  |  |  |
| Kobelt, Sobocki et al. 2008 | Spain | CUA | Survey on work capacity, not further specified | Health care + societal | ankylosing spondylitis | 57 | IFX in double blind trial |  |  | IFX-no IFX | 24955 | -97618 | -122574 |
|  |  |  |  |  |  |  | IFX in Spanisch open trial |  |  | IFX-no IFX | 9823 | -259993 | -269817 |
| Kobelt et al. 2007 | UK | CUA | Survey on work capacity, not further specified | Health care | ankylosing spondylitis | 50 | IFX in Braun trial |  |  | IFX-no IFX | 55625 | -6184 | -61809 |
|  |  |  |  |  |  |  | IFX in ASSERT trial |  |  | IFX-no IFX | 53528 | -8141 | -61669 |
| Walsh et al. 2007 | Ireland | CMA | Time spent on treatment, average industrial wage | Health care | RA | 55 | IFX | 6.53% |  | IFX-ADA | 13383 | 17962 | 4579 |
|  |  |  |  |  |  |  | ADA | 8.92% |  |  |  |  |  |
| Spalding and Hay 2006 | USA | CUA | Average employment rates compared with RA employment rage, age-adjusted income, productivity data of patients + or – ETN | Health care | RA | 55-60 | MTX |  |  | ADA-MTX | NS | 53107 |  |
|  |  |  |  |  |  |  | ADA |  |  | ETN-MTX |  | 74763 |  |
|  |  |  |  |  |  |  | ETN |  |  | ADA+MTX-MTX |  | 1620556 |  |
|  |  |  |  |  |  |  | ADA + MTX |  |  | IFX+MTX-MTX |  | 341055 |  |
|  |  |  |  |  |  |  | IFX + MTX |  |  |  |  |  |  |
| Kobelt et al. 2006 | Canada | CUA | Lost work capacity. Incl. Early retirement, HCA | Health care | ankylosing spondylitis | NS | No IFX |  |  | IFX-no IFX | 32562 | 30626 | -1936 |
|  |  |  |  |  |  |  | IFX |  |  |  |  |  |  |
| Boonen et al. 2006 | NL | CUA | Sick leave in friction period, FCA + HCA | Societal | active ankylosing spondylitis | 42 | TAU | 72.24% | 8.64% | ETN-TAU | 126100 | 88730 | -37370 |
|  |  |  |  |  |  |  | ETN | 45.65% | 4.95% | FCA | 126100 | 129222 | 3122 |
|  |  |  |  |  |  |  | IFX |  |  | IFX-TAU | 206220 | 188982 | -17238 |
|  |  |  |  |  |  |  |  | 44.21% | 3.99% | FCA | 206220 | 209492 | 3272 |
| Kobelt, Andlin-Sobocki et al. 2004 | UK | CUA | Days absent, early retirement, sex-dependent hourly wages, HCA | Health care | ankylosing spondylitis | 57 | Pl |  |  | IFX-Pl | 141762 | 68585 | -73177 |
|  |  |  |  |  |  |  | IFX |  |  |  |  |  |  |
| Kobelt, Eberhardt et al. 2004 | Sweden | CUA | Days absent, average annual gross income, HCA | Societal | RA | 56.6 | ETN |  |  | IFX-ETN | 52355 | 51216 | -1139 |
|  |  |  |  |  |  |  | IFX |  |  |  |  |  |  |
| Kobelt et al. 2003 | UK+Sweden | CUA | Days absent + early retirement | UK: health care, Sweden: health care +societal | RA | 53.6 | Sweden 1 yr IFX + MTX | 77.17% |  | IFX+MTX-MTX | 33665 | 4098 | -29567 |
|  |  |  |  |  |  |  | Sweden 1 yr MTX | 82.89% |  |  |  |  |  |
|  |  |  |  |  |  |  | UK 1 yr IFX + MTX | 53.05% |  | IFX+MTX-MTX | 3254 | 2735 | -519 |
|  |  |  |  |  |  |  | UK 1 yr MTX | 65.64% |  |  |  |  |  |
| Wong et al. 2002 | USA | CUA | Indirect cost estimates first year trial based, for further years PC were estimated to be one or three times DC | Health care | RA | 52.6 | MTX | 62.68% |  | IFX+MTX-MTX | 39658 | 11457 | -28202 |
|  |  |  |  |  |  |  | IFX + MTX | 60.13% |  |  |  |  |  |
| Lidgren et al. 2008 | Sweden | CUA | HCA, not further specified | Societal | early breast cancer | 55 | TAU | 45.32% |  | Test 1-TAU | -63214 | 42342 | 105556 |
|  |  |  |  |  |  |  | IHC test 1 | 57.48% |  | Test 2-TAU | -46804 | 59233 | 106037 |
|  |  |  |  |  |  |  | IHC test 2 | 59.73% |  | Test 3-TAU | -46270 | 39837 | 86107 |
|  |  |  |  |  |  |  | IHC test 3 | 58.24% |  | Fish test-TAU | -42740 | 40387 | 83127 |
|  |  |  |  |  |  |  | Fish test | 58.56% |  |  |  |  |  |
| Norum et al. 2007 | Norway | CUA | Average % female in workforce, Employers annual labor costs during 17 visits | Health care + societal | breast cancer adjuvant | 50 | No TRAS 10% |  |  | TRAS-No TRAS 10% | 44907 | 22081 | -22826 |
|  |  |  |  |  |  |  | TRAS 10% |  |  |  |  |  |  |
|  |  |  |  |  |  |  | No TRAS 20% |  |  | TRAS-No TRAS 20% | 22453 | 11040 | -11413 |
|  |  |  |  |  |  |  | TRAS 20% |  |  |  |  |  |  |
| Norum & Holtmon 2005 | Norway | CMA | Estimated employment rate patients estimated by MDs and compared with average age-sex employment and income, FCA+HCA | Health care + societal | early breast cancer | 50 | FEC DI100 | 79.28% | 27.67% | FEC100-CMF100 | 328 | 328 | 0 |
|  |  |  |  |  |  |  | FEC DI90 | 80.19% | 28.81% | FEC90-CMF90 | 320 | 320 | 0 |
|  |  |  |  |  |  |  | FEC DI 80 | 79.35% | 30.05% | FEC80-CMF80 | -60 | -60 | 0 |
|  |  |  |  |  |  |  | CMF DI100, DI90, DI80 |  |  |  |  |  |  |
| Dewilde et al. 2006 | Sweden | CUA | NS | Societal | severe peristent IgE-mediated (allergic) asthma | 43 | TAU |  |  | OM-TAU | NS | 63568 |  |
|  |  |  |  |  |  |  | OM |  |  |  |  |  |  |
| Manns et al. 2002 | Canada | CUA | Estimated employment rates of ICU patients, average gross annual salary | Health care | severe sepsis | 61.1 | TAU | 1.61% |  | Act. P.C-TAU | 24134 | 23267 | 866 |
|  |  |  |  |  |  |  | Activated protein C | 1.62% |  |  |  |  |  |
| Kobelt et al. 2008 | Sweden | CUA | Short-time absence and early retirement | Societal | MS | 28.6 | TAU |  |  | NAT-TAU | 42397 | -12481 | -54879 |
|  |  |  |  |  |  |  | NAT |  |  |  |  |  |  |
| Gani et al. 2008 | UK | CUA | NS | Health care | highly active RRMS | 36 | NAT |  |  | NAT-Inferon β | NS | 3722 |  |
|  |  |  |  |  |  |  | Inferon β |  |  | NAT-GA |  | 3361 |  |
|  |  |  |  |  |  |  | Glatiramer acetate |  |  | NAT-BSC |  | 13645 |  |
|  |  |  |  |  |  |  | BSC |  |  |  |  |  |  |

**UK = United Kingdom, USA = United states of America, NL = the Netherlands, NS = not specified, FCA = friction cost approach, HCA = human capital approach**

**RA = Rheumatoid arthritis, MS = multiple sclerose. RRMS = Relapsing-Remitting multiple sclerose, HAQ = health assessment questionnaire**

**TAU = treatment as usual, IFX = infliximab, FEC = fluorouracil, epirubicin, cyclophosphamide, CMF = cyclophosphamide, methotrexate, fluorouracil, ADA = adalimumab, MTX = methotrexate, RTX = rituximab, ETN = etanercept, DMARD = disease-modifying antirheumatic drugs, IHC = immunohistochemical, XELOX = capecitabine/oxaliplatin, FOLFOX = 5-fluorouracil/leucovorin/oxaliplatin, IV/IV = outpatient intravenous paclitaxel and carboplatin, IV/IP = inpatient intravenous paclitaxel and intraperitoneal cisplatin plus outpatient intraperitoneal paclitaxel, TRAS = trastuzumab, OM = omalizumab, NAT = natalizumab, Act.P.C = activated protein C, Pl = placebo, DI = dose intensity, GA = glatiramer acetate, BSC = best supportive care, MDs = medical doctors, DC =direct costs**

References

Boonen, A., D. van der Heijde, J. L. Severens, A. Boendermaker, R. Landewé, J. Braun, J. Brandt, J. Sieper, and S. van der Linden. 2006. Markov model into the cost-utility over five years of etanercept and infliximab compared with usual care in patients with active ankylosing spondylitis. *Annals of the Rheumatic Diseases* 65 (2) (Feb-2006): 201-8.

Bristow, R. E., A. Santillan, R. Salani, T. P. Diaz-Montes, R. L. Giuntoli, B. C. Meisner, D. K. Armstrong, and K. D. Frick. 2007. Intraperitoneal cisplatin and paclitaxel versus intravenous carboplatin and paclitaxel chemotherapy for stage III ovarian cancer: A cost-effectiveness analysis. *Gynecologic Oncology* 106 (3) (Sep-2007): 476-81.

Davies, A., M. A. Cifaldi, O. G. Segurado, and M. H. Weisman. 2009. Cost-effectiveness of sequential therapy with tumor necrosis factor antagonists in early rheumatoid arthritis. *The Journal of Rheumatology* 36 (1) (Jan-2009): 16-26.

Dewilde, S., F. Turk, M. Tambour, and T. Sandström. 2006. The economic value of anti-IgE in severe persistent, IgE-mediated (allergic) asthma patients: Adaptation of INNOVATE to sweden. *Current Medical Research and Opinion* 22 (9) (Sep-2006): 1765-76.

Gani, R., G. Giovannoni, D. Bates, B. Kemball, S. Hughes, and J. Kerrigan. 2008. Cost-effectiveness analyses of natalizumab (tysabri) compared with other disease-modifying therapies for people with highly active relapsing-remitting multiple sclerosis in the UK. *PharmacoEconomics* 26 (7) (2008): 617-27.

Kobelt, G., P. Andlin-Sobocki, S. Brophy, L. Jönsson, A. Calin, and J. Braun. 2004. The burden of ankylosing spondylitis and the cost-effectiveness of treatment with infliximab (remicade). *Rheumatology (Oxford, England)* 43 (9) (Sep-2004): 1158-66.

Kobelt, G., P. Andlin-Sobocki, and W. P. Maksymowych. 2006. The cost-effectiveness of infliximab (remicade) in the treatment of ankylosing spondylitis in canada. *The Journal of Rheumatology* 33 (4) (Apr-2006): 732-40.

Kobelt, G., J. Berg, P. Lindgren, B. Jonsson, L. Stawiarz, and J. Hillert. 2008. Modeling the cost-effectiveness of a new treatment for MS (natalizumab) compared with current standard practice in sweden. *Multiple Sclerosis (Houndmills, Basingstoke, England)* 14 (5) (Jun-2008): 679-90.

Kobelt, G., K. Eberhardt, and P. Geborek. 2004. TNF inhibitors in the treatment of rheumatoid arthritis in clinical practice: Costs and outcomes in a follow up study of patients with RA treated with etanercept or infliximab in southern sweden. *Annals of the Rheumatic Diseases* 63 (1) (Jan-2004): 4-10.

Kobelt, G., L. Jönsson, A. Young, and K. Eberhardt. 2003. The cost-effectiveness of infliximab (remicade) in the treatment of rheumatoid arthritis in sweden and the united kingdom based on the ATTRACT study. *Rheumatology (Oxford, England)* 42 (2) (Feb-2003): 326-35.

Kobelt, G., P. Sobocki, J. Mulero, J. Gratacos, E. Collantes-Estevez, and J. Braun. 2008. The cost-effectiveness of infliximab in the treatment of ankylosing spondylitis in spain. comparison of clinical trial and clinical practice data. *Scandinavian Journal of Rheumatology* 37 (1): 62-71.

Kobelt, G., P. Sobocki, J. Sieper, and J. Braun. 2007. Comparison of the cost-effectiveness of infliximab in the treatment of ankylosing spondylitis in the united kingdom based on two different clinical trials. *International Journal of Technology Assessment in Health Care* 23 (3) (2007): 368-75.

Lidgren, M., B. Jönsson, C. Rehnberg, N. Willking, and J. Bergh. 2008. Cost-effectiveness of HER2 testing and 1-year adjuvant trastuzumab therapy for early breast cancer. *Annals of Oncology : Official Journal of the European Society for Medical Oncology / ESMO* 19 (3) (Mar-2008): 487-95.

Lindgren, P., P. Geborek, and G. Kobelt. 2009. Modeling the cost-effectiveness of treatment of rheumatoid arthritis with rituximab using registry data from southern sweden. *International Journal of Technology Assessment in Health Care* 25 (2) (2009): 181-9.

Maniadakis, N., V. Fragoulakis, D. Pectasides, and G. Fountzilas. 2009. XELOX versus FOLFOX6 as an adjuvant treatment in colorectal cancer: An economic analysis. *Current Medical Research and Opinion* 25 (3) (12-Feb-2009): 797-805.

Manns, B. J., H. Lee, C. J. Doig, D. Johnson, and C. Donaldson. 2002. An economic evaluation of activated protein C treatment for severe sepsis. *The New England Journal of Medicine* 347 (13) (26-Sep-2002): 993-1000.

Norum, J., and M. Holtmon. 2005. Adjuvant fluorouracil, epirubicin and cyclophosphamide in early breast cancer: Is it cost-effective? *Acta Oncologica (Stockholm, Sweden)* 44 (7) (27-Jun-1905): 735-41.

Norum, J., J. A. Olsen, E. A. Wist, and P. E. Lønning. 2007. Trastuzumab in adjuvant breast cancer therapy. A model based cost-effectiveness analysis. *Acta Oncologica (Stockholm, Sweden)* 46 (2) (2007): 153-64.

Spalding, J. R., and J. Hay. 2006. Cost effectiveness of tumour necrosis factor-alpha inhibitors as first-line agents in rheumatoid arthritis. *PharmacoEconomics* 24 (12) (2006): 1221-32.

van den Hout, W. B., Y. P. Goekoop-Ruiterman, C. F. Allaart, J. K. de Vries-Bouwstra, J. M. Hazes, P. J. Kerstens, D. van Zeben, et al. 2009. Cost-utility analysis of treatment strategies in patients with recent-onset rheumatoid arthritis. *Arthritis and Rheumatism* 61 (3) (15-Mar-2009): 291-9.

Walsh, C. A., P. Minnock, C. Slattery, N. Kennedy, F. Pang, D. J. Veale, B. Bresnihan, and O. FitzGerald. 2007. Quality of life and economic impact of switching from established infliximab therapy to adalimumab in patients with rheumatoid arthritis. *Rheumatology (Oxford, England)* 46 (7) (Jul-2007): 1148-52.

Wong, J. B., G. Singh, and A. Kavanaugh. 2002. Estimating the cost-effectiveness of 54 weeks of infliximab for rheumatoid arthritis. *The American Journal of Medicine* 113 (5) (1-Oct-2002): 400-8.
